# CellProfiler Analyst: interactive data exploration, analysis and classification of large biological image sets

**DOI:** 10.1093/bioinformatics/btw390

**Published:** 2016-06-26

**Authors:** David Dao, Adam N. Fraser, Jane Hung, Vebjorn Ljosa, Shantanu Singh, Anne E. Carpenter

**Affiliations:** ^1^ Imaging Platform, Broad Institute of Harvard and MIT, Cambridge, MA 02142, USA; ^2^ Department of Informatics, Technical University of Munich, Munich, Bavaria 80333, Germany; ^3^ Department of Chemical Engineering, Massachusetts Institute of Technology (MIT), Cambridge, MA 02139, USA

## Abstract

**Summary:** CellProfiler Analyst allows the exploration and visualization of image-based data, together with the classification of complex biological phenotypes, via an interactive user interface designed for biologists and data scientists. CellProfiler Analyst 2.0, completely rewritten in Python, builds on these features and adds enhanced supervised machine learning capabilities (Classifier), as well as visualization tools to overview an experiment (Plate Viewer and Image Gallery).

**Availability and Implementation:** CellProfiler Analyst 2.0 is free and open source, available at http://www.cellprofiler.org and from GitHub (https://github.com/CellProfiler/CellProfiler-Analyst) under the BSD license. It is available as a packaged application for Mac OS X and Microsoft Windows and can be compiled for Linux. We implemented an automatic build process that supports nightly updates and regular release cycles for the software.

**Contact:** anne@broadinstitute.org

**Supplementary information**: Supplementary data are available at *Bioinformatics* online.

## 1 Introduction

CellProfiler Analyst is open-source software for biological image-based classification, data exploration and visualization with an interactive user interface designed for biologists and data scientists. Using data from feature extraction software such as CellProfiler (Kamentsky *et al.*, 2011), CellProfiler Analyst offers easy-to-use tools for exploration and mining of image data, which is being generated in ever increasing amounts, particularly in high-content screens (HCS). Its tools can help identify complex and subtle phenotypes, improve quality control and provide single-cell and population-level information from experiments.

Some distinctive and critical features of CellProfiler Analyst are its user-friendly object-based machine learning interface, its ability to handle the tremendous scale of HCS experiments (millions of cell images), its gating capabilities that allow observing relationships among different data displays, and its exploration tools which enable interactively viewing connections between cell-level data and well-level data, and among raw images, processed/segmented images, extracted features and sample metadata.

Compared to other commonly-cited open-source biological image classification software like Ilastik (Sommer *et al.*, 2011), CellCognition (Held *et al.*, 2010) and WND-CHARM (Orlov *et al.*, 2008), CellProfiler Analyst has the advantage of containing companion visualization tools, being suitable for high-throughput datasets, having multiple classifier options, and allowing both cell and field-of-view classification. Advanced Cell Classifier (Horvath *et al.*, 2011) shares many of the classification features of CellProfiler Analyst, but it lacks HCS data exploration and visualization tools. Compared to command-line-based data exploration software like cellHTS (Boutros *et al.*, 2006) and imageHTS (Pau *et al.*, 2013) and the web tool web CellHTS2 (Pelz *et al.*, 2010), CellProfiler Analyst provides interactive object classification and image viewing. Several other software tools (e.g. the HCDC set of modules for KNIME (Berthold *et al.*, 2009)) are no longer available/maintained.

Here, we present major improvements to CellProfiler Analyst. Since its original publication (Jones *et al.*, 2008), CellProfiler Analyst has been rewritten in Python (vs. its original language, Java) with significant enhancements. While keeping the original functionality allowing researchers to visualize data through histograms, scatter plots and density plots and to explore and score phenotypes by sequential gating, the key new features include:

- multiple machine learning algorithms that can be trained to identify multiple phenotypes in single cells or whole fields of view, by simple drag and drop
- more efficient handling of large scale, high-dimensional data
- a gallery view to explore images in an experiment, and cells in individual images and
- a plate layout view to explore aggregated cell measurements or image thumbnails for single or multiple plates.

## 2 New features in CellProfiler Analyst 2.0

**Fig. 1.**
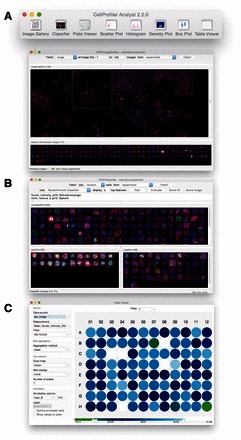
**User Interface of CellProfiler Analyst. (A**) Main Toolbar and Image Gallery; (**B**) Classifier; (**C**) Plate Viewer (Color version of this figure is available at *Bioinformatics* online.)

*Classifier:* CellProfiler Analyst 1.0 allowed researchers to train a single classifier (Gentle Boosting) to recognize a single phenotype (two-class) in individual cell images (rather than whole fields-of-view) (Jones *et al.*, 2009). In CellProfiler Analyst 2.0 (Fig. 1) , Classifier can perform cell and field-of-view-level classification of multiple phenotypes (multi-class) using popular models like Random Forest, SVM and AdaBoost from the high performance machine learning library scikit-learn (Pedregosa *et al.*, 2011), which yields a ∼200-fold improvement in speed (Supplementary Data 1). First, cell- or whole-image samples from the experiment are fetched and sorted by drag and drop into researcher-defined classes, making up the annotated training set. Fetching can be random, based on filters, based on per-class predictions of an already-trained classifier, or based on active learning. The new active learning option speeds annotation by presenting uncertain cases. In addition, researchers can view full images of each sample and drag and drop cells from the image for annotation. Next, a classifier is trained on this set. After training on the annotated set, a model’s performance can be evaluated by cross validation in the form of a confusion matrix and precision, recall and F1 score per class. The model can then be used to quantify cell phenotypes or whole-image phenotypes.

*Image Gallery:* CellProfiler Analyst 2.0 offers a convenient new Image Gallery tool (Fig. 1A), in addition to the existing visualization/exploration tools with standard plotting and gating capabilities in version 1.0 (Jones *et al.*, 2008). Image Gallery provides a convenient grid view allowing an overview of images. A variety of options are provided to filter images based on experiment-specific metadata, e.g. gene name, compound treatments, etc. Multiple filters can be combined to refine the search. Images can be displayed as a custom-sized thumbnail or in full resolution, and the color assigned to each channel in the image can be customized to highlight structures of interest. Individual segmented cells can be viewed for each image, and can be dragged and dropped into the Classifier window.

*Plate Viewer* **:** Many large-scale imaging experiments take place in multi-well plate format. Researchers are often interested in seeing their data overlaid on this format, to check for systematic sample quality issues, or to see results from controls placed in particular locations, at a glance. The Plate Viewer tool (Fig. 1C) displays aggregated and/or filtered measurements (according to customizable color maps) or a thumbnail image for each well. Automatically imported annotations can be viewed, and individual annotations can be manually added or deleted for each well.

*Additional features* **:** Additional features added to CellProfiler Analyst vs. version 1.0 have been described elsewhere, such as Tracer, a tool that complements the object tracking functionality of CellProfiler, including visualization and editing of tracks (Bray and Carpenter, 2015), as well as workspaces for saving progress and display settings across sessions (Bray *et al.*, 2012). The website, manual and tutorials have been redesigned and updated to the new version.

## 3 Future directions

The redesigned CellProfiler Analyst contains useful classification and visualization features in an interactive interface that facilitates data analysis and exploration of biological images. Its code base forms a solid foundation for integrating new classifiers into the tool, potentially including deep learning architectures. We also intend to integrate methods for constructing per-sample ‘profiles’ from raw morphological measurements to support morphological profiling applications (Caicedo *et al.*, 2016; Bray *et al.*, 2016).

## Supplementary Material

Supplementary Data
