# Supplementary material for: CellProfiler Analyst: interactive data exploration, analysis and classification of large biological image sets
Source: Bioinformatics. 2016 Jun 26;32(20):3210–2. doi: 10.1093/bioinformatics/btw390 (PMC5048071; doi:10.1093/bioinformatics/btw390)
Supplement: Supplementary Data [file btw390_supplementary_data.zip › Supplementary_Text_1.pdf]

Introduction

CellProfiler Analyst (CPA) provides tools for classifying biological images and exploring and visualizing multidimensional data (particularly from high-throughput experiments) that has been extracted from companion image analysis software CellProfiler.

CellProfiler Analyst’s primary tools:

- **Image Gallery** displays (full and cell) images with a variety of filter options and can be used interactively with other tools.
- **Classifier** enables cell and field-of-view-level classification of multiple phenotypes using popular supervised machine learning models.
- **Plate Viewer** displays data according to the spatial layout of the experiment, such as a multi-well plate or microarray.
- **Scatter Plot**, **Histogram**, and **Density Plot** display numerical data.
- **Table Viewer** displays numerical and text data in a spreadsheet format, where data points can be clicked to display images.
- **Normalization Tool** creates a new datatable with normalized and feature-selected columns.

The CellProfiler project is based at the Broad Institute Imaging Platform. It was started by Anne E. Carpenter and Thouis (Ray) Jones in the laboratories of David M. Sabatini and Polina Golland at the Whitehead Institute for Biomedical Research and MIT's CSAIL. David Dao currently leads the development of CellProfiler Analyst. CellProfiler Analyst is tested on MacOS 10.10 and 10.11, Windows 7 and 10, and to a lesser extent Linux. Running the software from Linux currently requires use of the source code, which is beyond the scope of this document.

CellProfiler Analyst is distributed under the BSD-3 Clause (see LICENSE.txt).

I. Preliminary data requirements

CPA requires access to the following data sources:

- An image table and an object table containing measurements and metadata

    These may reside in a MySQL or SQLite database or in a set of comma-separated value (CSV) files. A MySQL database is recommended, though you may need to consult with your local information technology staff to set up a database server. See section II.B for more information. The tables must contain a few datacolumns needed by CellProfiler Analyst to access images and data properly, such as an Image ID column to link the per-image and per-object tables, file path and file name columns to specify where images are stored, and X, Y location columns to specify where each object resides within the image. These configuration details are specified in a properties file. Note: if image classification is specified in the properties file, an object table is not required. See section III.

- The images that were analyzed to generate the above-mentioned Table Viewers

    These can be stored either locally or remotely and accessed via HTTP. The directory structure does not matter as long as the file paths stored in the image table point to the correct images. Throughout CPA, the term image is meant to include all image data associated with an analyzed field-of-view. An image in this sense usually includes several individual monochromatic images that show the different wavelengths (channels) as well as images that show outlines of identified objects. You can specify any number of image channels (including, for example, outlines of objects that resulted from image processing) by adding path and filename columns to the image table of your database for each channel. CPA currently requires image files to be monochromatic; several individual channels can be combined into a color image for viewing within the software. CPA currently supports the following image file types: BMP, CUR, DCX, Cellomics DIB, FLI, FLC, FPX, GBR, GD, GIF, ICO, IM, IMT,

IPTC/NAA, JPG/JPEG, MCIDAS, MIC, MSP, PCD, PCX, PIXAR, PNG, PPM, PSD, SGI, SPIDER, TGA, TIF/TIFF, WAL, XBM, XPM, XV Thumbnails.

> ***Note***
>
> While designed for high-throughput, image-based biological experiments, CellProfiler Analyst is also useful for the exploration of other multi-dimensional data sets, particularly when data points are linked to images.

I.A Example image table

The image table requires one column for a unique image ID and a pair of columns for each channel represented in the images: one column for the image path, and one column for the image file name (which may include some part of the path to the image, such as the subdirectory that contains the file). These columns do not need to have specific names; you will indicate which column names correspond to image ID, image path, and image filename when configuring the properties file. The remaining columns can contain measurements and metadata about each image.

> ***Note***
>
> While MySQL and SQLite support diverse column names, CPA will not handle column names that contain commas. In general, we advise that you use only alphanumeric characters and underscores in the names of your table columns.

An image table for an experiment involving cells imaged for GFP and Hoechst would have two channels and would look something like this:

ImageNumber	Image_FileName_OrigCY3	Image_PathName_OrigCY3	Image_FileName_OrigCY5	Image_PathName_OrigCY5	Metadata_Plate	Metadata_Well
1	Capture 8 -CY3.tif	/imaging/analysis/2009...	Capture 8 -CY5.tif	/imaging/analysis/2009...	3	H07
2	Capture 9 -CY3.tif	/imaging/analysis/2009...	Capture 9 -CY5.tif	/imaging/analysis/2009...	3	H07
3	Capture 1 -CY3.tif	/imaging/analysis/2009...	Capture 1 -CY5.tif	/imaging/analysis/2009...	3	H08
4	Capture 10 -CY3.tif	/imaging/analysis/2009...	Capture 10 -CY5.tif	/imaging/analysis/2009...	3	H08
5	Capture 2 -CY3.tif	/imaging/analysis/2009...	Capture 2 -CY5.tif	/imaging/analysis/2009...	3	H08
6	Capture 3 -CY3.tif	/imaging/analysis/2009...	Capture 3 -CY5.tif	/imaging/analysis/2009...	3	H08

I.B Example object table

The object table requires four columns: a foreign key image ID column that corresponds to the image ID in the image table, a unique object ID column, a column for the object x-location, and a column for the object y-location. CPA expects the location columns to correspond to the x-y pixel coordinates of the objects' centroids; the corresponding column names that are produced by CellProfiler depend on the name of the objects; for example, if nuclei were measured, the column names would be Nuclei_Location_Center_X and Nuclei_Location_Center_Y. Again, these columns do not need to have specific names; you indicate which column names correspond to these functionalities when configuring the properties file. Additional columns in this table typically contain measurements for each object, but are completely up to the user.

> ***Note***
>
> While MySQL and SQLite support diverse column names, CPA will not handle column names that contain commas. In general, we advise that you use only alphanumeric characters and underscores in the names of your table columns.

An object table for an experiment involving cells imaged for GFP and Hoechst would have two channels and would look something like this:

ImageNumber	ObjectNumber	MitoCells_Location_Center_X	MitoCells_Location_Center_Y	Appro	ApproxMitoC	ApproxMito
1	1	21.1326	39.1226	11	21.1326	39.1226
1	2	380.944	103.364	19	380.944	103.364
1	3	191.364	140.127	23	191.364	140.127
1	4	677.998	194.763	27	18	153.459
1	5	464.76	205.278	36	15.59	178.15
1	6	401.093	226.286	40	677.998	194.763
1	7	57.1001	231.304	42	464.76	205.278
1	8	191.526	283.919	46	401.093	226.286

II. Installation and getting started

CPA releases

All CellProfiler-Analyst releases can be found here

Prerequisites

Install JDK 1.8 and Java

II.A Mac OS X

Open dmg file and copy CellProfiler-Analyst.app to /Applications.

II.B Windows 7

Run the setup.exe to create the executable and shortcuts.

II.C Using the example dataset

Download the CPA example dataset from http://cellprofiler.org/ or here and unzip it to create the cpa_example directory. This directory contains:

1. example.properties - Configuration file for CPA (see section III).
2. MyTrainingSet.txt - Example training set file to be used in the Classifier (see section V).
3. images/ - Images from the screen used in the example.
4. per_image.csv - Comma Separated Values file for image data. This file was exported by CellProfiler’s ExportToDatabase module.
5. per_object.csv - Comma Separated Values file for object data. This file was exported by CellProfiler’s ExportToDatabase module.
6. example_SETUP.SQL - Used by CPA to create an internal database (SQLite). It can also be used to create a MySQL database. This file was exported by CellProfiler’s ExportToDatabase module.

Run the CPAnalyst file created by the install process above. A dialog will appear asking you to select a properties file. Navigate to the cpa_example directory and select the example.properties file. You're now ready to experiment with CellProfiler Analyst!

III. Setting up the properties file

The properties file is a plain text file that contains the configuration information necessary for CPA to access your data and images. This file can be stored anywhere on your computer. It is selected and loaded upon startup of CPA.

If you use CellProfiler to produce the data to be analyzed in CPA, you can automatically generate a nearly complete properties file with, using the ExportToDatabase module. Otherwise, you can create one manually, referring to the Properties_README or the example provided below as a template.

Each setting in the properties file is stored on a separate line in the form field = value(s), and the order of the settings is not important. Lines that begin with a # are ignored by CPA and may be used for comments. Settings that require a file path may be specified either as absolute or relative to the directory that the properties file is found in.

Note: When editing the properties file, it is important to use an editor that is capable of saving plain text. CPA cannot read files of type .doc, .rtf, etc. We suggest using Notepad on Windows, TextEdit on Mac OS, and Emacs on Linux.

Note: CPA 2.0 is not compatible with properties files from CellProfiler Analyst version 1.0, but the two formats may be easily converted by hand. Contact us on the CellProfiler forums if you need help with this.

Note: All fields described in the sections below (after the properties file example) are required unless explicitly described as "optional." In your own properties file, you would replace values surrounded with <> with the relevant information.

III.A Properties file example

```
==== CPA 2.0 properties file for example dataset ====
# ==== Database Info ====
db_type     = sqlite
db_sql_file = example_SETUP.SQL
# ==== Table info ====
image_table  = per_image
object_table = per_object
# ==== Column info ====
image_id   = ImageNumber
object_id  = ObjectNumber
plate_id   = plate
well_id    = well
cell_x_loc = Nuclei_Location_CenterX
cell_y_loc = Nuclei_Location_CenterY
# ==== Image access info ====
image_url_prepend =
# ==== Image Path and Filename Columns ====
image_path_cols = Image_Pathnames_Path_Origdna,
  Image_Pathnames_Path_Origdna, Image_Pathnames_Path_Origdna
image_file_cols = Image_Filenames_Filename_origActin,
  Image_Filenames_Filename_origpH3, Image_Filenames_Filename_origdna
image_names = Actin, pH3, DNA
image_channel_colors = red, green, blue
# ==== Global Meta data ====
object_name = cell, cells
plate_type  = 96
# ==== Classify ====
classifier_ignore_columns = Nuclei_Location.*, Meta.*,
# ==== Other ====
image_tile_size = 40
```

III.B Database access values

These settings tell CPA how to access your database. One of the 4 modes below may be used. Include only the settings listed for that mode of storage in your properties file.

To connect to a MySQL database

A MySQL database is recommended for storing extremely large data that may need to be accessed from different computers. CellProfiler can readily create a MySQL database if a database server is available; see the instructions for the ExportToDatabase module for details.

```
db_type    =  mysql
db_port    =  3306
db_host    =  your host name
db_name    =  your database name
db_user    =  your user name
db_passwd  =  your password
```

To connect to an SQLite database

SQLite is another mode of data storage, in which tables are stored in a large, database-like file on your local computer rather than a database server. This is easier to set up than a full- featured MySQL database and is at least as fast, but it is not a good choice of storage if the data is to be accessed by multiple concurrent connections. This mode of storage also puts a limitation on the number of rules used in the FastGentleBoosting Algorithm in Classifier. CellProfiler is able to export data in this format using the ExportToDatabase module.

```
db_type         =  sqlite
db_sqlite_file  =  path and filename of SQLite db file
```

To access multiple comma-separated value (.csv) files produced by CellProfiler

This mode tells CPA to find your image and object tables in several CSV files produced using CellProfiler's ExportToDatabase module. In this mode of data storage, you specify the path to the xxx_SETUP.SQL file written by CellProfiler's ExportToDatabase module (the "xxx" will be the prefix you specified in the ExportToDatabase module settings). CPA will then use this file to find the CSV files produced by ExportToDatabase to create an SQLite database file in your home directory. This could take a long time for larger databases, but only needs to be done once. Note: This feature expects the CSV files to be in the same directory as the SQL file. If they are renamed or moved apart from the SQL file, CPA will not find them and won't be able to construct your database.

```
db_type      =  sqlite
db_sql_file  =  path and filename of .SQL file from ExportToDatabase
To access data stored in two CSV files
```

This mode tells CPA to find your image and object tables in two CSV files. When you run CPA with these settings, it looks for column headers in the first row of each file and inserts the data into an SQLite database file in your home directory. Again, this could take a long time for larger databases, but only needs to be done once.

```
db_type          =  sqlite
image_csv_file   =  path and filename of image csv
object_csv_file  =  path and filename of object csv
```

III.C Database Structure Values

Database table names

The following fields identify the image and object tables:

```
image_table   =  name of the table with one row per image
object_table  =  name of the table with one row per object
```

Database column names

The following fields identify the columns that specify unique image and object numbers:

```
image_id   =  image_number key column from the image & object table
object_id  =  object_number key column from the object table
```

The following field (optional) identifies the columns that specify a table number, which is useful in cases where multiple image tables have been manually merged and the image number column is no longer unique:

```
table_id  =  table_number key column from the image & object tables
```

The following fields identify the columns in the object table that specify the x and y location of each object within the original image:

```
cell_x_loc  =  object x-location column from the object table
cell_y_loc  =  object y-location column from the object table
```

The following fields (optional) identify plate and well columns in your image table and are only required by the PlateMapBrowser tool:

```
plate_id  =  plate_id column from the image table
well_id   =  well_id column from the image table
```

III.D Image access values

The following fields specify the columns in the image table that contain the image paths and file names. CPA can access images stored locally or via HTTP. In either case, the URLs (locations) of images must be specified in the image table. You can specify any number of image channels, separated by commas:

```
image_path_cols = 1st channel image path column, 2nd channel image path column,...
image_file_cols = 1st channel image file column, 2nd channel image file column,...
```

The following fields (optional) allow you to provide a common name for each of the above channels and to specify a default display color for each. If you don’t assign these, channels will appear as Channel0, Channel1, etc. in CPA and they will be assigned default colors. Channel colors can later be changed while using CPA (section III.D.1). Valid colors are red, green, blue, magenta, cyan, yellow, gray, none. Follow the same order of channels as for image_path_cols and image_file_cols:

```
image_names  = Actin, pH3, DNA, ...
image_channel_colors = red, green, blue, ...
```

The following field (optional) specifies how to combine each channel when displaying an image. Valid blend mode values are add and subtract. By default, additive blending is used, which is best for combining channels from fluorescent images. However, subtractive blending may be desirable when you wish to display outlines over a brightfield image. In this case, the brightfield channel is added while the outline channel is subtracted. The result would be the normal brightfield image with black outlines created from subtracting the white outline values from the white background of the brightfield image.

```
image_channel_blend_modes = add, add, add, ...
```

The following field allows you to specify how many channels should be read from each image file specified by image_path_cols and image_file_cols. By default, CPA will expect to find 1 channel per image. If you wish to read 3 channels from a single RGB image, you would set: channels_per_image = 3. Remember that image_names, image_channel_colors, and image_channel_blend_modes will then each need 3 values specified instead of just 1.

```
channels_per_image  = 1, 1, 1, ...
```

The following field allows you to specify a URL prefix to add to all image paths. That is, CPA will place the contents of this field in front of the path and filename values extracted from image_path_cols and image_file_cols. This is especially useful if you wish to move your images to another location without having to change the image_path_cols column in the database, or when the images are stored remotely and need to be accessed through HTTP. For example, if you set image_url_prepend = http://yourserver.com/images and the path and file name in the database for a given image are yourpath and file.png, then CPA will open http://yourserver.com/images/yourpath/file.png.

```
image_url_prepend  =  http://yourserver.com/images
```

The following field specifies the crop size of the object tiles, that is, the pixel size of the square “window” that shows an individual object in certain tools within CPA. A good rule of thumb is to set this value to be slightly larger than the approximate diameter of the objects:

```
image_tile_size  =  tile size in pixels
```

III.F Other optional settings

Metadata

```
object_name = singular name, plural name
```

The following field tells PlateMapBrowser what size plates were used. Supported types are 96, 384, 1536, or 5600 (for cell microarrays)

```
plate_type  =  number of wells
```

Columns ignored by Classifier

The following field (optional) specifies columns in the per-object table that you want Classifier to ignore when classifying objects. Classifier automatically ignores columns defined by the table_id, image_id, and object_id fields, as well as any columns that contain non-numeric data. When using this field to specify additional columns to ignore, note that your column names cannot contain commas because commas are used to separate each column name. You can also use regular expressions to refer to a set of column names for Classifier to ignore:

```
classifier_ignore_columns = column name or regular expression A,
  column name or regular expression B
```

For example, the following statement will ignore the column named WellID, any columns whose name contains the text Red (case-sensitive), and any columns that end in _Position.

```
classifier_ignore_columns = WellID, .*Red.*, .*_Position
```

Classifier default training set

The following field (optional) specifies the full path and file name for a training set that Classifier should automatically load when it is launched:

```
training_set =  your directory/your subdirectory/your file
```

Area-based scoring

The following field (optional) specifies a column in your per-object table that will be summed and reported when scoring with Classifier. You can use this, for example, to report the total area of objects in each class, in addition to the number of objects in each class (which is Classifier's default output):

```
area_scoring_column = your alternate column name
```

Outputting per-object classes

The following field (optional) specifies a MySQL table name for Classifier to create in your database when Score All is used. The class name of each object will be written to this table along with image and object IDs so it may be linked back into your existing tables. Warning: If this table name already exists, it will be overwritten:

```
class_table = your class table name
```

Checking your tables

The following field (optional) tells Classifier whether to check your tables at startup for anomalies such as missing column indices (an index is a database structure that greatly improves the speed of data retrieval) or orphaned objects (objects that do not correspond to an image in the image table). The operation can take up to a minute if your object table is extremely large.

```
check_tables = yes or no
```

Different tumbnail size for Image Gallery

The following field (optional) tells Image Gallery, how large the thumbnail of the field of view should be. If nothing is set, image_size = image_tile_size

```
image_size = 200
```

III.G Advanced settings

These more advanced settings require some knowledge of SQL.

ImageFilter

The following fields (optional) specify a subset of the images in your experiment from which to fetch and score objects in Classifier. Multiple filters can be created by using the format below and replacing 'XXX' with a name for the filter. Names can include alphanumeric characters and underscores.

```
filter_SQL_XXX = SQL select statement that returns
 \ image-keys for image you want to include
```

For example, here is a filter that returns only images from plate 1:

```
filter_SQL_Plate_1 = SELECT ImageNumber
 \ FROM per_image WHERE plate="1"
```

This example returns only images from with a gene column that starts with CDK:

```
filter_SQL_CDKs = SELECT ImageNumber FROM per_image WHERE gene REGEXP 'CDK.*'
```

This example does the same for a database that includes a table_id column TableNumber:

```
filter_SQL_CDKs = SELECT ImageNumber, TableNumber
  \ FROM per_image WHERE gene REGEXP 'CDK.*'
```

Filters may be created from within CPA by choosing the option “*create new filter*”. A dialog box will appear that allows you to define filters on the fly. First choose a name for your filter, then choose the column you would like to filter and specify the constraints. For example, below we select lonely those images from well A07:

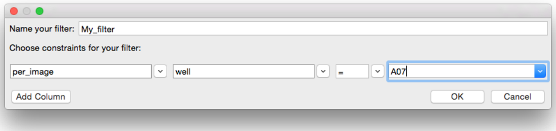

You may select more than one column for your filter. For example if you would like only those images from well A07 which have more than 10 cells, you would click ‘Add Column’ and specify the filter as such:

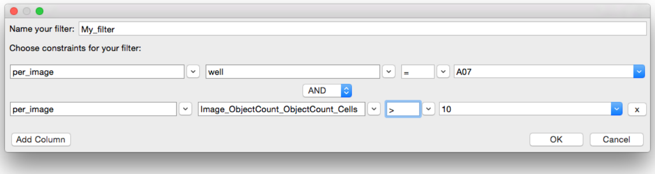

When you have completed your filter, click OK. The filter will now be available to you to use in the CPA tools for the remainder of your session.

Groups

You can define ways of grouping your image data (for example, when several images represent the same experimental sample), by linking column(s) that identify unique images (the image- key) to another set of columns (the group-key, which may reside in other tables, provided the tables have a common key). Grouping is useful, for example, when you want to aggregate counts for each class of object and their scores on a per-group basis (e.g.: per-well) instead of on a per-image basis when scoring with Classifier. It will also provide new options in the Classifier fetch “from” menu so you can fetch objects from images with specific values for the group columns. Multiple groups can be created by replacing 'XXX' with the desired group name:

```
group_SQL_XXX = MySQL SELECT statement that returns image-key
 \ columns followed by group-key columns
```

For example, if you wanted to be able to group your data by unique plate names, you could define a group called SQL_Plate as follows:

```
group_SQL_Plate = SELECT ImageNumber, plate FROM per_image
```

As another example, you could define a way to group unique wells:

```
group_SQL_Well+Plate = SELECT ImageNumber, plate,
 \ well FROM per_image
```

The previous example, but for a database including a table_id column TableNumber:

```
group_SQL_Well+Plate = SELECT TableNumber,
 \ ImageNumber, plate, well FROM per_image
```

Image Classification

CellProfiler Analyst 2.2 also supports classification directly from image features. To allow this, the biologist has to set the classification type flag to image

```
classification_type = image
```

IV. The CellProfiler Analyst Interface

To run CPA, double-click the CPAnalyst.exe file on Windows or CPAnalyst.app on Mac OS. Once you have selected a properties file to load, the following small window will appear.

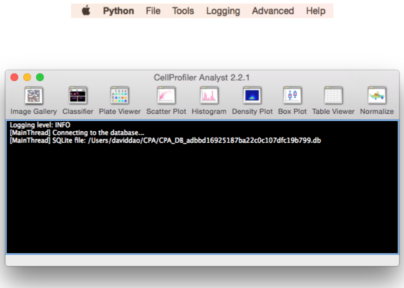

CellProfiler Analyst main window.

This window provides a toolbar at the top with icons to launch each of CPA's tools. The currently available tools are Image Gallery, Classifier, Plate Viewer, Scatter Plot, Histogram, Density Plot and Table Viewer. These tools can also be launched from the Tools menu. More tools will be added to this suite in the future.

Below the toolbar is a logging console that all the tools will use to provide feedback on status, warnings, etc. This console can be configured to display messages at 5 levels of verbosity chosen from the Logging menu. In order of increasing verbosity, these are:

- Critical: Virtually no messages. Will only report critical errors.
- Errors: Only reports error messages.
- Warnings: Only reports warnings and error messages.
- Info: Reports general status information as well as any warnings and errors.
- Debug: Reports technical information such as SQL queries in addition to all other messages listed above.

You can save the log to a file by selecting File > Save Log, or copied by selecting the text and using Ctrl+C (or your operating system default copy command).

You will also find menu items to save and load workspaces under the File menu. See Section XIII for more on workspaces in CPA and how they can help you analyze new data more quickly.

SQL query tool

The advanced menu provides a SQL query tool for writing SQL statements to query the DB

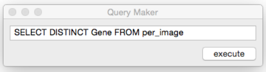

Querying for all unique genes in per_image table

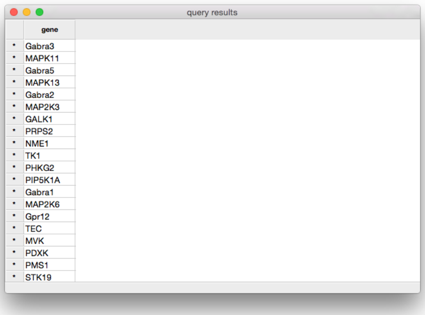

Result of the query above for cpa_example dataset

V. Classifier

Classifier allows you to train the computer to identify objects of interest by applying iterative, user-supervised machine learning methods to object measurements.

You first request (**Fetch**) object tiles (cropped from their original images), then manually sort them into classification bins (representing object classes), to form an annotated set. Once each bin contains several example objects, you can start training Classifier on the annotated set, i.e., asking the machine to learn how to differentiate the classes. Once Classifier is trained, you can continue training by fetching and sorting either more random objects or those objects that Classifier scores as being in a particular class; by fetching the objects predicted by Classifier to belong to a certain class and correcting errors in these classifications, subsequent Classifiers rapidly improve. Usually several rounds of refinement are necessary to train Classifier to recognize the classes of interest.

Once classification reaches a desirable accuracy, Classifier can “score” your experiment. This entails classifying all objects, counting how many objects of each class are in each image or group (if you have defined groups in your properties file; see section II.G), and computing the enrichment/depletion of each class per image or per group.

V.A Classifier quick-start guide

1. Launch Classifier and enter the number of objects you want Classifier to fetch.
2. Specify whether Classifier should select these objects from the entire experiment, a single image or a group, and whether it should apply any filters. Groups will only be available if defined in your properties file (section II).
3. Click **Fetch**. Objects will appear in the unclassified bin.
4. Manually sort the unclassified objects into classification bins, adding additional bins if needed. Often, two bins are used: **positive** and **negative**. Bin names can be changed by right clicking empty space in the relevant bin.
5. Enter the number of top features you want to see (or if FastGentleBoosting is the chosen classifier, the maximum number of rules you want Classifier to look for). Click **Train**.
6. Repeat steps 2–5 to fetch and sort more objects. You will be able to specify that Classifier only retrieves objects that it deems to be in a particular class or objects that are difficult to classify so that you can correct errors.

7. Click **Score Image** to visualize object classifications in a particular image (you will be asked to enter an image ID number). Objects can be dragged and dropped into bins from the **Image Viewer** or **Image Gallery** for further training.
8. It is important to save the training set for future refinement, to re-generate scores, and as a record of your experiment. It is advisable to do so before proceeding to scoring your experiment since scoring may take a long time for large screens. Select File > **Save Training Set** from the menu bar (or `ctrl+S`).
9. Click **Score All** to have Classifier score your entire experiment (optionally with groups or filters). Classifier will present the results in a Table Viewer (described in section IV).
10. You can click on column headings to sort the data by that column, helping you identify images that are highly enriched in a given object class, or images that simply have a high count of those objects. You can double-click the headers of rows to view the corresponding images and then drag and drop objects from the resulting image(s) into classification bins to improve the classifier.
11. You can save Classifier's scores for each image (or group) from the Table Viewer using File > **Save data to CSV** or **Save per-image counts** to CSV to create comma-separated value files. You can also view the scores with CPA's visualization tool, Plate Viewer, by using **Database > Write Temporary Table in Database** and running the Plate Viewer (section IV).

V.B In-depth guidance for using Classifier

V.B.1 Configuring Classifier

Launch Classifier by clicking the Classifier icon in the CPA toolbar. The main Classifier screen will appear. If you have previously saved a training set, you can load it using **File > Load Training Set:**

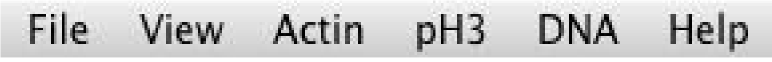

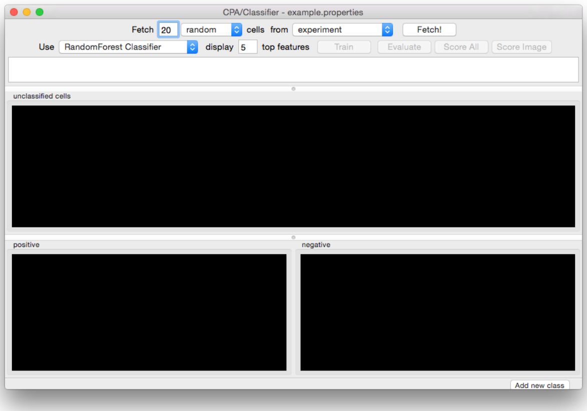

Initial Classifier screen.

Adding, Deleting, and Renaming Bins

- **Tip:** Use as few bins as necessary for the relevant downstream analysis; adding too many bins can decrease the overall accuracy.

To add more bins at any time, click the **Add new class** button in the extreme lower right-hand corner of the window. You will see the **Rename class** popup window:

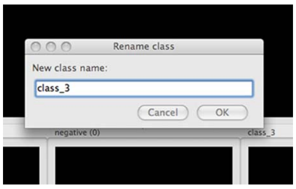

Adding a sorting bin.

Right-clicking inside any bin displays a popup menu that contains a number of options, including **deleting** and **renaming** bins. The remaining options in this menu apply to the *contents* of the 17 bin. See section III.C.3 for more information.

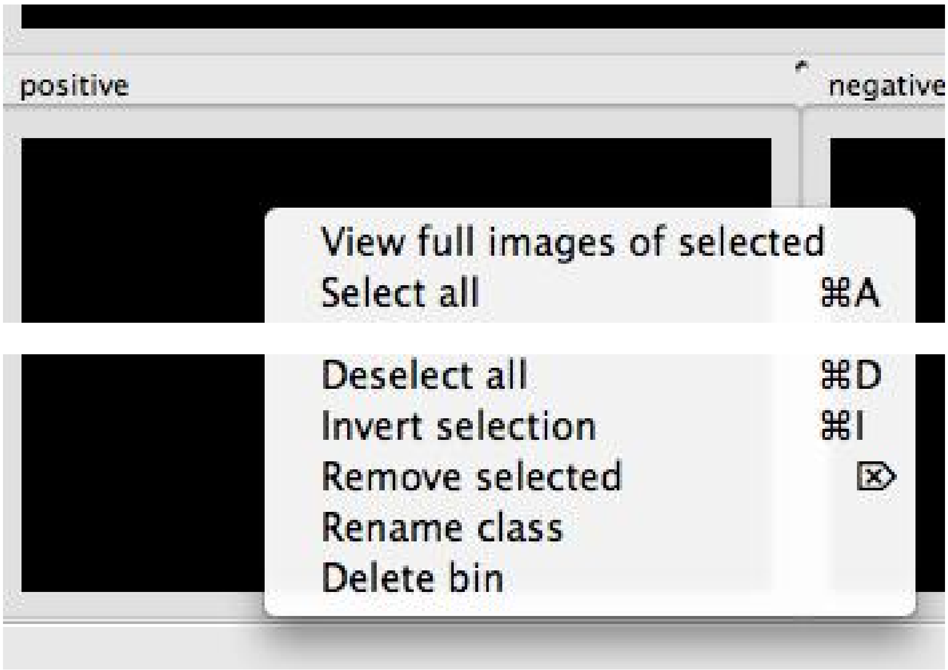

Right clicking on a sorting bin.

Adjusting the display

The menu bar at the top of the screen contains options for adjusting the display of the image tiles that will be displayed. **View > Image Controls** will bring up the same control panel found in the **Image Viewer** tool (section V), and the **channel menus** can be used to map different colors onto the respective channels. (**Actin**, **pH3**, and **DNA**, in this example; named so in the properties file as described in section II.)

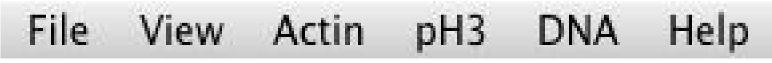

Classifier menu bar.

V.B.2 Fetch an initial batch of objects

Objects are fetched (retrieved) using the top portion of the main Classifier window:

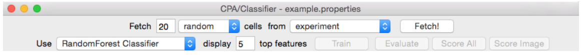

Controls to fetch objects.

How many objects? Enter the number of objects you want Classifier to fetch (default = **20**)

Which class of objects should be retrieved? At this stage, **random** will be the only option available in the left-hand menu. After you **Train** Classifier (section III.C.5, following), new options will appear relating to each classification bin.

From which images? Two system-supplied default values in the right-hand menu are **experiment** and **image**. Select **experiment** to have Classifier retrieve objects from your entire experiment; select **image** to retrieve objects from a particular image (you will be asked to type its ID number). If you want to fetch objects from particular subsets of images in the experiment (e.g., control samples), you can set up filters by choosing the third default value in the right-hand menu **create new filter**; you can also define filters and groups of images in your properties file (described earlier in section II.G).

Click the **Fetch** button (located next to the right-hand menu) when you are ready to proceed, and you will see results like this:

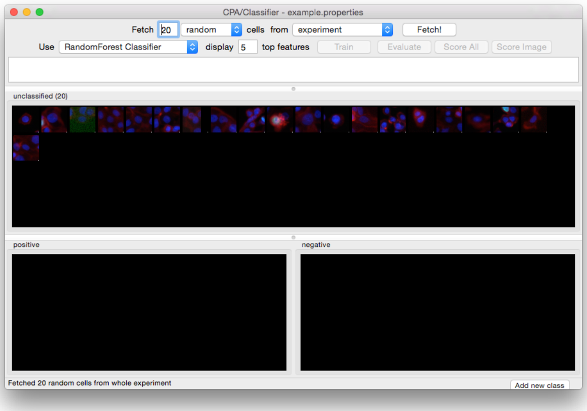

Twenty unclassified cells have been fetched and are ready for initial sorting.

V.B.3 Sort the initial batch of objects

Use your mouse to drag and drop object tiles into the classification bins you configured in step III.C.1. If you are uncertain about the classification of a particular object, it can be ignored or removed by selecting it and pushing the Delete key. Keep in mind, however, that classifier will ultimately score ALL objects found in your table unless you define filters to ignore certain images (see section II.F).

Important: A small dot is displayed in the center of each tile as your mouse hovers over it. The object that falls under this dot is the object that must be sorted. In the example below, the tile under the mouse should be sorted based on the blue cell underneath the dot, NOT the cells surrounding it. To change cropping size of the tile "window", adjust the field `image_tile_size` in the properties file (section II.D).

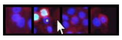

The object to be sorted is indicated by a small dot.

Once you have placed tiles in at least two bins, you have created Classifier's initial **training set**, which will be used to train the classifier to differentiate objects in different classes.

- **Tip:** Clicking on a tile will select it. Holding shift will allow you to add and remove tiles from the selection. All the tiles in a selection can be moved at once by dragging one of them to another bin.

Some helpful tools are available when you right-click on a tile:

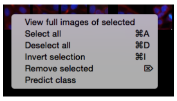

Right-clicking on a tile.

- Select **View full images of selected** or double-click an individual tile to show the object in the context of the image from which it was drawn. This launches the **Image Viewer** tool (section V).

Tip: Objects can dragged and dropped from the **Image Viewer** or **Image Gallery** into class bins just as they are from the bins themselves. Use `Shift+click` to add/remove multiple objects to/from a selection, and `ctrl+A/Ctrl+D` to select/deselect all objects in the image.

- **Select all/Deselect all** (`ctrl+A/ctrl+D`) selects/deselects all tiles in the bin so they can be dragged and dropped together.
- **Invert selection** (`ctrl+I`) to invert your selection (that is, select all non-selected tiles in the current bin and deselect all selected tiles).
- **Remove selected** (`Delete`) removes the selected tiles from the current bin.

V.B.4 Saving and loading training sets

Objects sorted into the bins are known as the **training set**. You can save the training set at any time, allowing you to close CPA and pick up where you left off later by re-loading the training set. Save and load training sets using **File > Save training set** or **File > Load training set**.

Warning: Loading a training set will cause all existing bins and tiles to be cleared.

V.B.5 Training Classifier

Continue repeating the process of fetching objects, sorting them into their appropriate classes, and training. Scoring (section III.C.6, following) can be used when you have finished creating a **training set** (that is, you are satisfied by its performance), but note that, as described later, scoring can also be used as another iterative step in creating the training set.

Assessing accuracy

The most accurate way to gauge Classifier's performance is to fetch a large number of objects of a given class (e.g., positive) from the whole experiment. The fraction of the retrieved objects correctly matching the requested phenotype indicates the classifier's general performance. For example, if you fetch 100 positive objects but find upon inspection that 5 of the retrieved objects are not positives, then you can expect Classifier to have a positive predictive value of 95% on individual cells (and similarly for negative predictive value in the case of two classes). Note that sensitivity, specificity, and negative and positive predictive values must be interpreted in the context of the actual prevalence of individual phenotypes, which may be difficult to assess a priori.

The **Evaluate** button calculates cross-validation metrics given the annotated set. Values closer to 1 indicate better performance. The cross-validation is 5 fold, and for each fold, the annotated set is split into a training and testing set (the split is stratified, meaning class proportions remain intact) and the algorithm is trained on the training set, then evaluated on the test set. To get final values, the evaluations are averaged over all folds. The evaluation can display a classification report, which is the recall, precision, and F1 score per class, or a confusion matrix, which is a matrix where the element in row i, column j has true class i and predicted class j.

Another way to gauge the classifier's performance is to use the **Score Image** button on positive and negative controls (see the following section). **Score Image** allows you to see qualitatively how Classifier performs on a single image. Although the results cannot be reliably extrapolated to other images, it can be useful to examine control images and further refine the classifier by adding misclassified objects in those images to the proper bins.

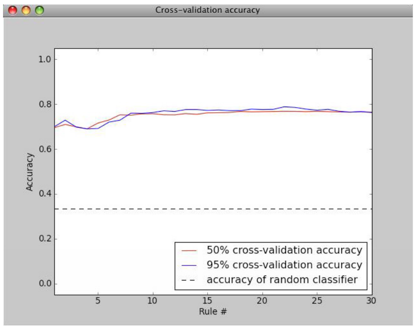

The relationship between accuracy on individual cells versus performance scoring wells for follow-up is complicated, because false positive and false negatives are not evenly distributed throughout an experiment. In practice, improving accuracy on individual cells leads to better accuracy on wells, and in general, the accuracy on wells is better than the per-cell accuracy.

V.B.6 Scoring

Score image

Scoring a single image can be useful in several ways:

- You can display an image and rapidly identify and correct classification errors in the image, by dragging and dropping objects from the image into bins.
- You can use it as visual feedback to verify your classifier's accuracy on a given image (especially a control image) at any point in the training process.
- You can also use it to check Classifier's classifications for individual images with unusual scores displayed in the Table Viewer produced by **Score All** (described in the next section).

To score a single image qualitatively, select **Score Image** and enter an image number. Classifier displays the image in **Image Viewer** (described in section V), with objects marked according to their classifications, based on the trained classifier. To save the resulting image as either a .jpg or .png file, select **File > Save Image** from the menu bar (or shortcut **Ctrl+S**).

> ## *Note*
>
> **Note**: This function is not yet capable of saving the classification markings.

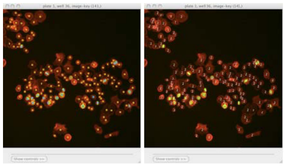

Scoring an image: Identifying classes by color (blue and yellow squares, left) and by number (right). Note that we have chosen to hide the blue channel (DNA stain) while viewing these images.

To display the object classes by number rather than color, select **View > View object classes as numbers** from the menu bar.

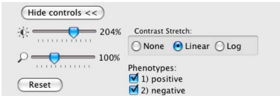

The Image Viewer control panel after scoring an image.

The brightness, contrast, and zoom controls work exactly as described for **Image Viewer** (section V). Note, however, the two checkboxes under **Phenotypes**: you can now select/deselect **positive** and **negative** results to display or hide only these objects in the image as requested.

Score all

Click **Score All** to classify all objects in your database using the current trained classifier. It can be helpful to score all images in the experiment and open some of the top-scoring images with **Score Image** to check classification accuracy. Training can be further refined by dragging and dropping objects from the image into bins in order to correct classification errors in images.

The result of **Score All** is a table of object counts and enrichment values for each classification you defined. You can then sort by these columns to find images (or groups, e.g., wells as collections of images) that are enriched or depleted for a particular classification, based on object counts or enrichment scores (see figure below for details).

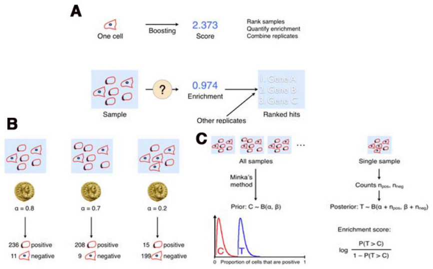

Description of enrichment score calculation. (A) While machine learning methods are used to produce per-cell scores, the challenge remains to model the sample distributions to generate a per-sample enrichment score. (B) Samples with varied positive/negative counts can be viewed as being drawn from a Beta distribution. (C) The full population is treated as independent samples to yield C = Beta(■, ■) which is used as the full-population-level prior for future observations. This prior is updated with new observations by computing the distribution of the positive fraction as the posterior T = Beta(■ + npos, ■ + nneg), where npos and nneg are the positive and negative counts, respectively. The enrichment score for each sample is then calculated as the logit of P(T > C).

Note: *Enrichment* scores are computed for each sample as the logit area under the ROC curve for the prior versus the posterior distribution. The prior is computed from the full experiment using a Dirichlet-Multinomial distribution (a multi-class extension of Beta-Binomial) fit to the groups, and the posterior is computed for each group independently; that is, each phenotype is treated as positive and all others as negative for each phenotype in turn. - **Tip**: In most cases results should be ranked by enrichment score because this score takes into account both the number of objects in the class of interest as well as the total number of objects in the group.

If you have defined any groups or filters, you will have the option to select them here for use in scoring. If no groups or filters are defined, the window will contain only the default group **Image** and the default filter **None**.

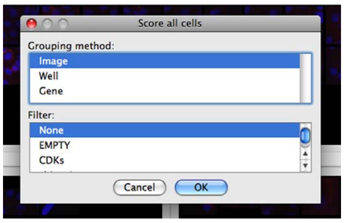

Classifier group/filter selection window.

Classifier presents its results in the **Table Viewer** tool, described in the next section. The table shows object counts and enrichment values for each phenotype you trained Classifier to recognize. To view this information graphically, return to the main Classifier screen and select **Tools > Plate Viewer** from the menu bar (see section VI for details).

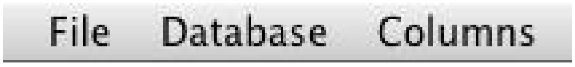

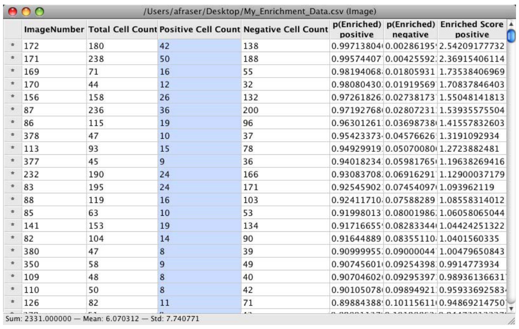

	ImageNumber	Total Cell Count	Positive Cell Count	Negative Cell Count	p(Enriched) positive	p(Enriched) negative	Enriched Score positive
*	172	180	42	138	0.99713804	0.00286195	2.54209177732
*	171	238	50	188	0.99574407	0.00425592	2.36915406114
*	169	71	16	55	0.98194068	0.01805931	1.73538406969
*	170	44	12	32	0.98080430	0.01919569	1.70837846403
*	156	158	26	132	0.97261826	0.02738173	1.55048141813
*	87	236	36	200	0.97192768	0.02807231	1.53935575504
*	86	115	19	96	0.96301261	0.03698738	1.41557832603
*	378	47	10	37	0.95423373	0.04576626	1.3191092934
*	113	93	15	78	0.94929919	0.05070080	1.2723882481
*	377	45	9	36	0.94018234	0.05981765	1.19638269416
*	232	190	24	166	0.93083708	0.06916291	1.12900037179
*	83	195	24	171	0.92545902	0.07454097	1.093962119
*	88	119	16	103	0.92411710	0.07588289	1.08558314012
*	85	63	10	53	0.91998013	0.08001986	1.06058065044
*	141	153	19	134	0.91716655	0.08283344	1.04424251322
*	82	104	14	90	0.91644889	0.08355110	1.0401560335
*	380	47	8	39	0.90999955	0.09000044	1.00479650843
*	350	58	9	49	0.90745601	0.09254398	0.9914773934
*	109	48	8	40	0.90704602	0.09295397	0.98936136631
*	110	50	8	42	0.90105078	0.09894921	0.95933692583
*	126	82	11	71	0.89884388	0.10115611	0.94869214750

Enrichment Table Viewer produced by Classifier. Here we have grouped the counts and statistics on a per-image basis. We have ordered the data by the "Enriched Score Positive" column. The most highly enriched images were 172, 171, 169, and 170. With the "Positive Cell Count" column selected, we can see in the status bar that there are a total of 2331 positive cells in our experiment, with a mean of 6.07 positive cells per image, and a standard deviation of 7.74.

XII. Table Viewer

Certain tools, such as Classifier, produce a table that will be opened in the Table Viewer. As well, any CSV file or database table can be viewed by using this tool.

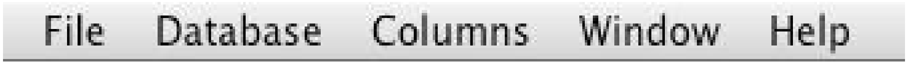

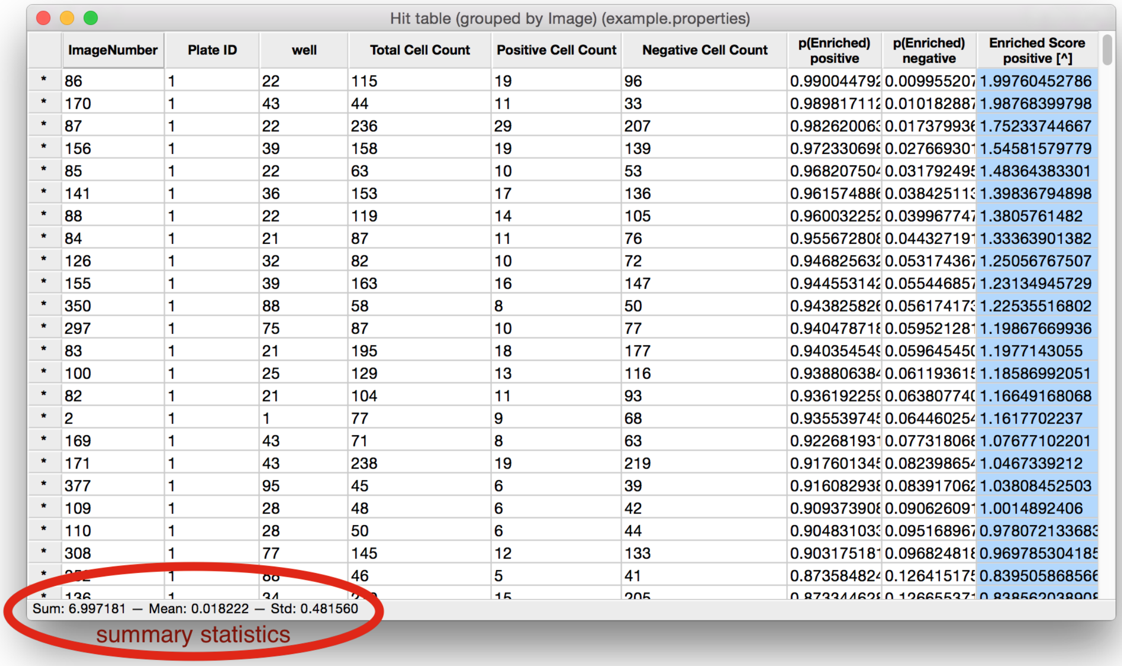

	ImageNumber	Plate ID	well	Total Cell Count	Positive Cell Count	Negative Cell Count	p(Enriched) positive	p(Enriched) negative	Enriched Score positive [^]
*	86	1	22	115	19	96	0.99004479	0.00995520	1.99760452786
*	170	1	43	44	11	33	0.98981711	0.01018288	1.98768399798
*	87	1	22	236	29	207	0.98262006	0.01737993	1.75233744667
*	156	1	39	158	19	139	0.97233069	0.02766930	1.54581579779
*	85	1	22	63	10	53	0.96820750	0.03179249	1.48364383301
*	141	1	36	153	17	136	0.96157488	0.03842511	1.39836794898
*	88	1	22	119	14	105	0.96003225	0.03996774	1.3805761482
*	84	1	21	87	11	76	0.95567280	0.04432719	1.33363901382
*	126	1	32	82	10	72	0.94682563	0.05317436	1.25056767507
*	155	1	39	163	16	147	0.94455314	0.05544685	1.23134945729
*	350	1	88	58	8	50	0.94382582	0.05617417	1.22535516802
*	297	1	75	87	10	77	0.94047871	0.05952128	1.19867669936
*	83	1	21	195	18	177	0.94035454	0.05964545	1.1977143055
*	100	1	25	129	13	116	0.93880638	0.06119361	1.18586992051
*	82	1	21	104	11	93	0.93619225	0.06380774	1.16649168068
*	2	1	1	77	9	68	0.93553974	0.06446025	1.1617702237
*	169	1	43	71	8	63	0.92268193	0.07731806	1.07677102201
*	171	1	43	238	19	219	0.91760134	0.08239865	1.0467339212
*	377	1	95	45	6	39	0.91608293	0.08391706	1.03808452503
*	109	1	28	48	6	42	0.90937390	0.09062609	1.0014892406
*	110	1	28	50	6	44	0.90483103	0.09516896	0.97807213368
*	308	1	77	145	12	133	0.90317518	0.09682481	0.96978530418
*	[illegible]	1	[illegible]	46	5	41	0.87358482	0.12641517	0.83950586856

Table Viewer produced by Classifier, grouped by well number and gene (only positive Enrichment Score column).

Features available in the Table Viewer

- **Sort**: Click on any column heading to sort the data based on that column. Click again to reverse the sort. To sort by more than one column (i.e., sort first by column X, then by column Y), click on the first heading of the first column you want to sort by, then hold shift and click on the next column heading. You can add any number of columns to a sorting in this way. Sorting order and direction is indicated in brackets at the end of the header name by a number and an arrow (^ or v). Clicking on any column header that is already part of a sorting will reverse the direction of the sort. Pressing shift+click on a column header that is already part of a sorting will remove that column from the sorting.
- **Show images**: Double-click on a row header to show the image or images in that row's group. Right-click to see a list of the image-keys in that row and click one to open it.
- **View summary statistics**: Click anywhere within the table to select a column. A summary of statistical information about the selected column(s) is displayed in the status bar at the bottom of the window. You may select multiple columns by pressing ctrl+click (cmd+click on a Mac).
- **Show/hide columns**: To select which columns are shown in the Table Viewer, select View > Show/hide columns and a dialog will appear with a list of columns. Check off the columns that you wish to show, and uncheck the column that you want hidden.
- **Saving and loading CSV files**: CPA can read and write CSV (comma-separated value) files by selecting File > Load table from CSV or File > Save table to CSV. When saving a table it's important to note that columns that were hidden (see above) will not be written to the CSV.
- **Saving and Loading database tables**: Table Viewer can also load and save database tables. This means you can easily open a CSV file in Table Viewer and then store that table in your database for plotting and analysis in CPA. Conversely, you can load a database table into Table Viewer (such as your per-object table), and save it on your computer as a CSV. As with CSVs, saving and loading from the database can be done via File > Load table from database and File > Save table to database. Note, when saving a table to the database, you will be prompted whether to store the table permanently or "for this session only" – which means the table will be removed from the database when you close CPA.

> ***Note***
>
> When saving a table to the database, you will be prompted whether to store the table permanently or "for this session only" – which means the table will be removed from the database when you close CPA.

VII. Image Viewer

Certain CPA tools, such as **Classifier**, display images in **Image Viewer**, and any image in the experiment can also be opened within CPA by choosing this tool from CPA's **Tools** menu or by clicking the **Image Viewer** icon in the toolbar.

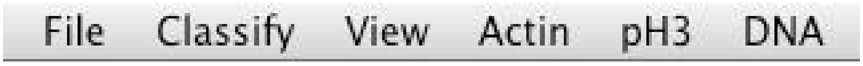

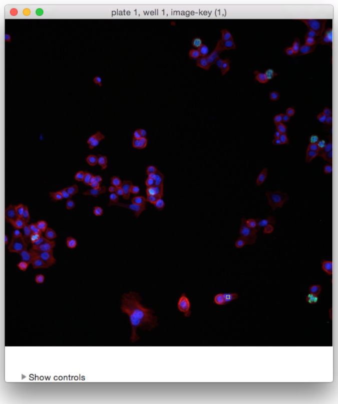

The Image Viewer.

When you open Image Viewer from CPA, it will prompt for the ID number of an image to show. If you also specified a `table_id` column in your properties file, then you will also be prompted for the table number. You can load additional images by selecting **File > Open Image (ctrl+O)**.

If you specified `plate_id` and `well_id` in your properties file, then Image Viewer will display the plate and well in the title of the window along with the image-key, which is image 328 in the example above.

The `image_names` that are specified in your properties file will appear in the menu bar. In the example above, the user has specified names for the **Actin**, **pH3**, and **DNA** channels. If you have not defined names for any channels, the menu bar will contain **channel-1**, **channel-2**, etc.

Click on one of the channel names to see a dropdown list of available colors for each – clicking on a color will map that color onto the channel. If you specified `image_channel_colors` in your properties file, those colors specified there should appear under each channel menu by default.

The Classifier menu provides an item that will let you classify the objects in the current image. This item is only enabled if Classifier is running and trained. For more on this feature see the **Score image** discussion in section III.C.6.

- **Tip**: You can use keyboard shortcuts to toggle each channel on/off: Ctrl+1, +2, +3, … will toggle first, second, third, etc. color channels.

Click **Show controls** (or press the space bar) to display the Image Viewer control panel, which controls brightness, contrast, and scale. This same control panel is available in Classifier by choosing **View > Image Controls** (see section III.C.1).

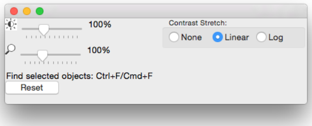

Image controls

The upper slider controls brightness; the lower one rescales the size of the image or image tiles. The **Contrast Stretch** controls let you stretch the image contrast by rescaling the pixel intensities of each channel independently to fit the range 0-1 (where 0 is unsaturated and 1 is saturated).

- **Linear** scales the image intensities linearly. For an image in the range 0-0.5, pixels at 0.5 intensity would be rescaled to 1.0, and pixels at 0.4 rescaled to 0.8; in short, all pixel intensities in the image would be doubled.

- **Log** transform scales the image intensities logarithmically, which has the effect of scaling dimmer pixels by a greater factor than it scales brighter pixels.

Warning: scaling in this way may yield misleading results. For example, if a channel of an image shows very little staining, the raw image may have intensities in a low, narrow range, e.g., from [0-0.01]. Stretching in this case would have the effect of raising the background intensity levels so that the image overall appears very bright.

VIII. Plate Viewer

Plate Viewer is a tool for browsing image-based data laid out on multi-well plates common to high-throughput biological screens. Supported formats are:

- **96 well plate** — 8 rows x 12 columns
- **384 well plate** — 16 rows x 24 columns
- **1536 well plate** — 32 rows x 48 columns
- **5600 spot microarray** — 40 rows x 140 columns

To launch this tool click the Plate Viewer icon in the CPA toolbar or select **Tools > Plate Viewer** from the CPA menu bar.

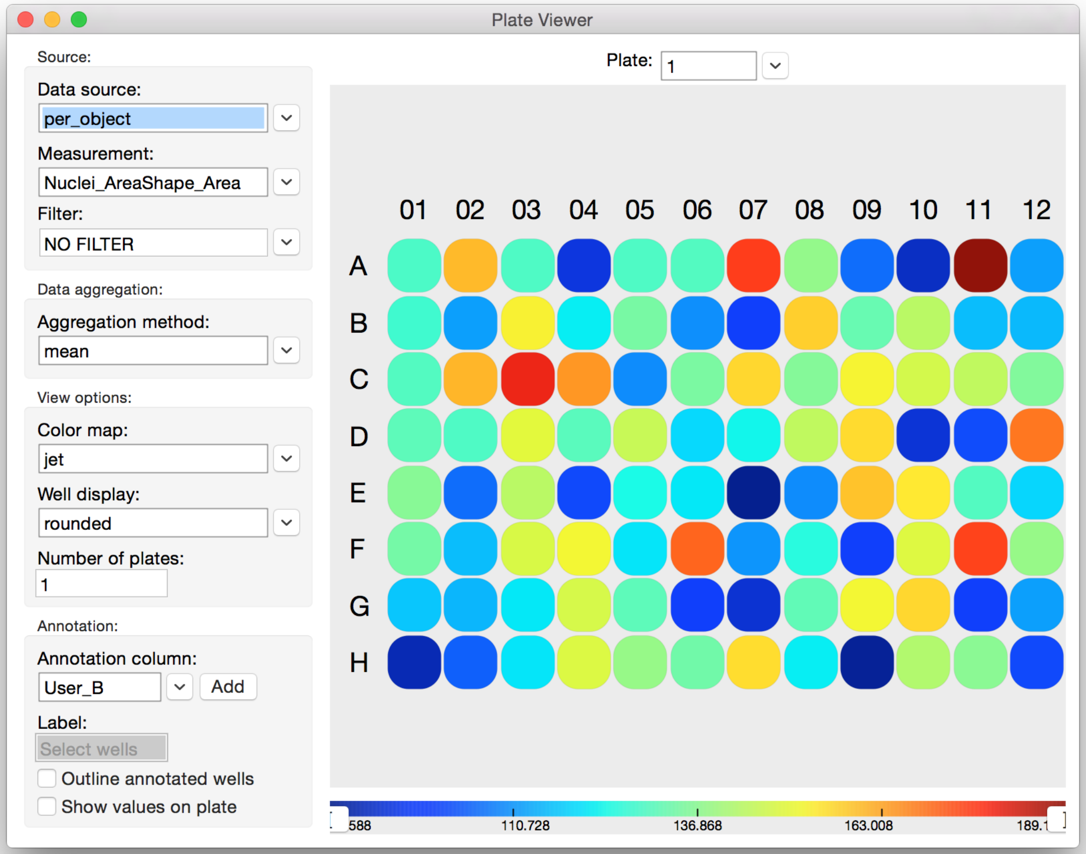

Note

An **X** in one of the wells means there was no data in the database corresponding to that particular well. However, it may still contain images that can be viewed, as described below.

In the colored plate display, right-clicking on a well will display a list of image-keys found in that well. Selecting one image-key will open that image in the **Image Viewer** (section V). Doubleclicking on a well will open all images from that well, each in its own Image Viewer.

Above the plate is a menu that lets you specify the plate number to display. Holding the cursor over a particular well will display a tooltip showing the value pertaining to that well.

The left-hand column contains many options for generating different views into your data.

- **Data source**: Allows you to select a table for visualization. To load fresh data from a CSV file directly into the Plate Viewer, select **File > Load CSV** from the menu bar at the top of the screen.
- **Measurement**: The column from the selected table that you would like to visualize.
- **Aggregation Method**: Measurements must be aggregated to a single number for each well so that they can be represented by a color. Options are **mean**, **sum**, **median**, **standard deviation**, **cv%** (coefficient of variation), **minimum**, and **maximum**. If you're viewing `object_count` from the image table, for example, you might select **sum** to visualize the sum of the object counts for all images that constitute a well.

  > **Important**: Plate Viewer is agnostic with regard to the type of data it aggregates, so viewing columns from the image table will not cause the viewer to access any data from the object table. For example, a biological screen with 4 images (sites) per well may be analyzed in CellProfiler, and cell measurements may be aggregated to each image and output to the image table. One column grom the table, `Image_Intensity_DNA_Mean_ Intensity`, could be visualized in Plate Viewer and aggregated from each image to each well by, say, the **maximum** option. This could be mistakenly thought to be reporting the maximum DNA intensity value of any cell from that well, when it is actually reporting the maximum of the mean per-image DNA intensity per-well.

- **Color Map**: Each value computed by the Aggregation Method is mapped to a color via a color map. The currently selected color map is represented in a bar beneath the plate maps. More than 50 color maps are available.
- **Well Shape**: Mostly for presentation purposes, you can select from different well shapes. Options are **square**, **rounded**, **circle**, and **image**.

  > Important: The "*image*" feature is still under development. This will display a rescaled image from each well in the place of the well itself. This can take a very long time to refresh since full sized images must be loaded for every well in view. For it to be useful, viewing 1 plate at a time and maximizing the window is advised. Note: that the color map is irrelevant when using this view.

- **Number of Plates**: Enter the number of plates you would like to view at once, and press the Enter or Return key.

Here is a four-plate view of the same experiment shown in the previous example, in which the user has chosen to view the mean of the per-object measurement *Spindle_AreaShape_Area* in each well across 4 plates.

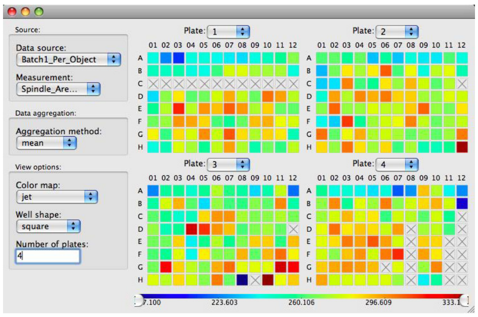

Viewing four 96-well plates at once in Plate Viewer. Here we are visualizing the mean Spindle_AreaShape_Area in each well. This makes it easy to visualize many thousands of object measurements in a meaningful way.

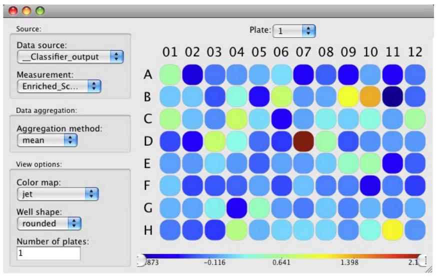

Viewing the sums of the per-object measurement tubulin_AreaShape_Area in each well of a single 384-well plate in Plate Viewer. Notice that while well C01 has the largest tubulin area (62406) in this plate, the color bar indicates that the largest value of this measurement in the experiment is 91617, which must be in some other plate.

The color bar axis at the bottom of the window shows how the aggregated values of the selected measurement column map to colors:

- The numbers at the far left and right of the axis represent the minimum and maximum values found in the entire experiment.
- The value range of the current plate(s) extends to the point where the color bar stops and a thin black line begins.

To assist in visualizing data, particularly when there are extreme outlier data points, you can **rescale** or **clip** the color bar by dragging the handles found at the far ends of the bar.

- **Color rescaling**: This will rescale the full color map to fit within the minimum and maximum values at the handles. Values below the range are mapped to the minimum (leftmost) color, values above the range are mapped to the maximum (rightmost) color.
- **Color clipping**: As with color rescaling, this will map values below the range to the minimum color, and values above the range to the maximum color. However, the full range of colors will not be rescaled to fit within the handles, it is cropped or “clipped.”

To change the mode from rescaling to clipping, right click on the bar and select **Value bracketing: CLIP**, to change it back to rescaling, do the same and select **Value bracketing: RESCALE**. To set the sliders back at the global min and max, select **Reset sliders**.

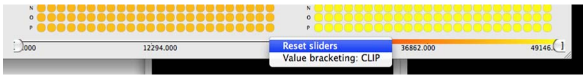

Selecting Color Bar options.

IX. Scatter Plot

Launch **Scatter Plot** from the main CPA interface by clicking the button in the toolbar. This tool will allow you create scatter plots from the numeric columns in your tables.

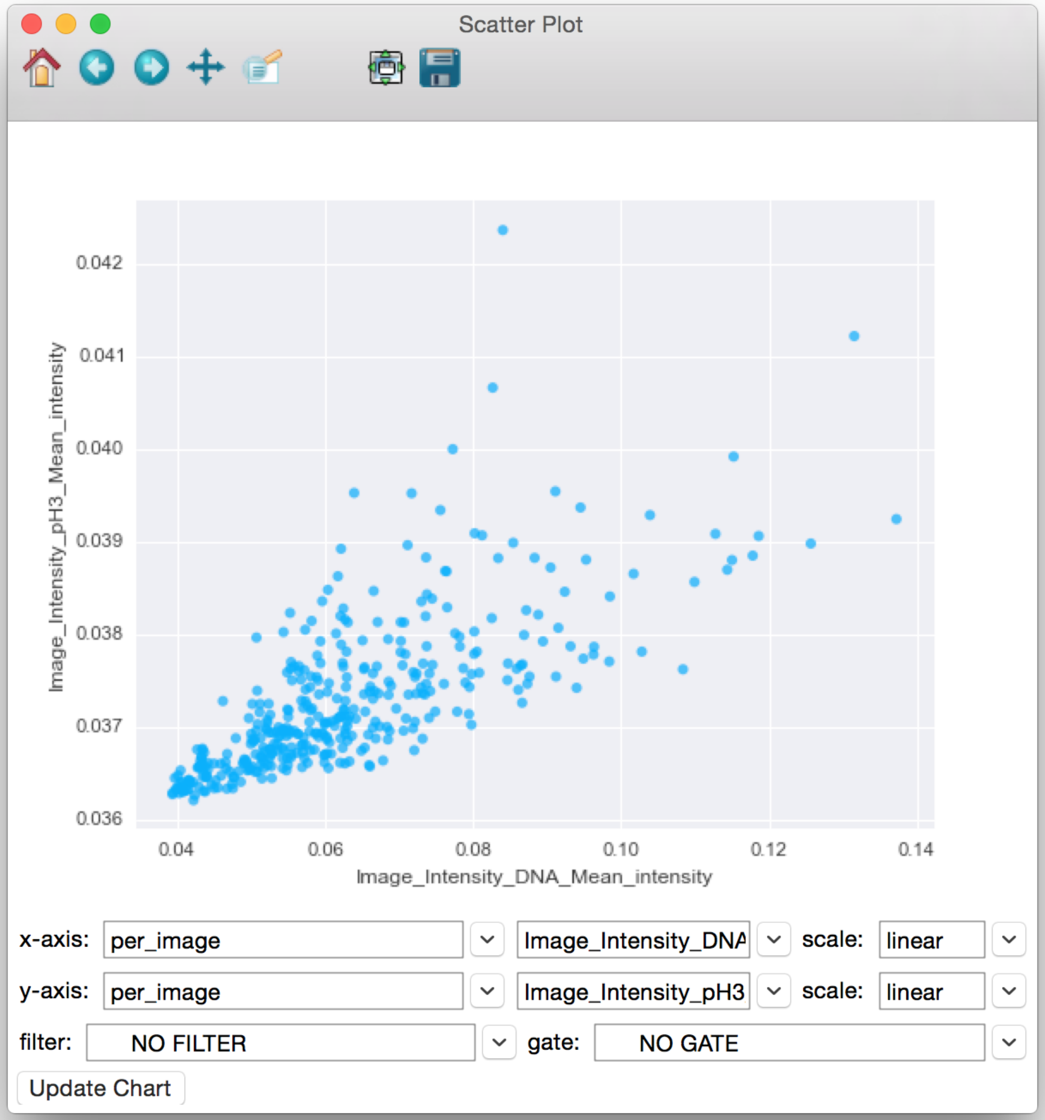

Using Scatter Plot to visualize the relationship between the Image_Intensity_DNA_Mean_intensity and the Image_Intensity_pH3_Mean_intensity image measurements.

To use the **Scatter Plot**, simply select the table whose columns you would like to plot from the table dropdown. Then select measurements from that table in the x-axis and y-axis dropdowns (only numeric columns will be available). To view an axis in the log scale, choose **log** from the x-scale or y-scale dropdown. The filter dropdown can be used to select filters defined in your properties file to limit the points being plotted. Finally, click the **Update Chart** button to view the plot.

> ***Note***
>
> Update may take a long time or even fail for large databases. We are working to improve this performance in later releases.

Once data is plotted, you can use the tools provided in the toolbar at the top of the window to 37 explore the plot. Going backwards from right to left, the tools are as follows:

- **Save**: Clicking this will give you choices for saving the plot as an image.
- **Zoom-to-rect**: This tool may be toggled on and off. When it is on, you can use the mouse to click and drag a rectangle to zoom in on. The extents of the rectangle will become the new extents of the axes. Use the Back button (below) to zoom back out.
- **Pan**: This tool may also be toggled. When on, you can use the mouse to pan the axes by clicking and dragging.
- **Forward** and **Back**: These 2 buttons will redo and undo the zooming and panning actions that you make
- **Home**: This will reset the view to the way it was after Update Chart was pressed

Selection: When the pan and zoom tools are toggled off, the default action of the mouse is to draw a freeform selection. You can add to an existing selection by holding the shift key while drawing a new shape. Likewise, you can subtract from a selection by holding alt and drawing a selection around the points you wish to deselect.

Viewing Images: You can view the images from a selection by right-clicking on the plot and selecting “Show images from selection” from the resultant popup menu. This will pop up a list of the selected image keys along with their plate and well information (if specified in your properties file). Double clicking on an image entry will launch that image in the **Image Viewer** tool.

X. Histogram Plot

Launch **Histogram Plot** from the main CPA interface by clicking the button in the toolbar. This tool allows you to create histogram plots from the numeric columns in your tables.

To use the **Histogram Plot**, simply select the table whose columns you would like to plot from the table dropdown. Then select a single measurement from that table in the x-axis dropdown (only numeric columns will be available). You can also enter the number of bins to use. The filter dropdown can be used to select filters defined in your properties file to limit the points being plotted. Finally, click the **Update Chart** button to view the plot.

Once data is plotted, you can use the tools provided in the toolbar at the top of the window to explore the plot. Going backwards from right to left, the tools are as follows:

- **Save**: Clicking this will give you choices for saving the plot as an image.
- **Zoom-to-rect**: This tool may be toggled on and off. When it is on, you can use the mouse to click and drag a rectangle to zoom in on. The extents of the rectangle will become the new extents of the axes. Use the Back button (below) to zoom back out.
- **Pan**: This tool may also be toggled. When on, you can use the mouse to pan the axes by clicking and dragging.
- **Forward** and **Back**: These 2 buttons will redo and undo the zooming and panning actions 38 that you make
- **Home**: This will reset the view to the way it was after Update Chart was pressed.

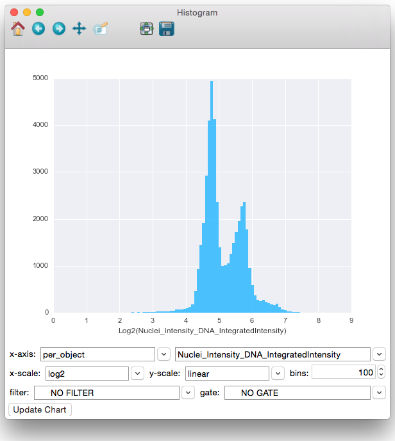

Using the Histogram Plot to view the distribution of the Nuclei_Intensity_DNA_IntegratedIntensity measurement, with the x-axis displayed in the log space. The distribution appears to be bimodal, each peak representing an accumulation of cells in different stages of mitosis.

XI. Density Plot

Launch **Density Plot** from the main CPA interface by clicking the button in the toolbar. This tool will allow you create density plots from the numeric columns in your tables.

To use the **Density Plot**, simply select the table whose columns you would like to plot from the table dropdown. Then select the measurements you would like to plot from that table in the xaxis and y-axis dropdowns (only numeric columns will be available). To view an axis in the log scale, choose **log** from the x-scale or y-scale dropdown. You may also enter the grid size to use for binning the data. The grid size is similar to the bins input in the histogram plot except that it 39 is applied along both dimensions, so a grid size of 50 would yield 50x50 points. The filter dropdown can be used to select filters defined in your properties file to limit the points being plotted. Finally, click the **Update Chart** button to view the plot. With the plot displayed, you can change the color map by selecting different maps from the color map dropdown.

Once data is plotted, you can use the tools provided in the toolbar at the top of the window to explore the plot. Going backwards from right to left, the tools are as follows:

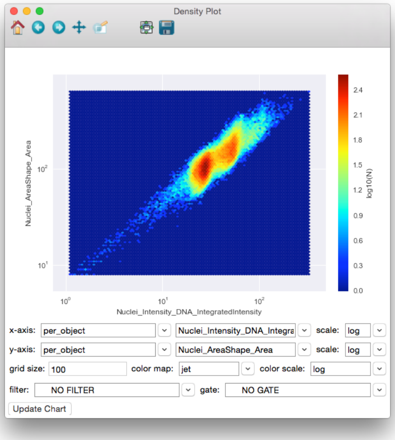

A density plot of Nuclei_Intensity_DNA_IntegratedIntensity versus Nuclei_AreaShape_Area with log axes, log color scale, and a grid size of 100.

• **Save**: Clicking this will give you choices for saving the plot as an image.

- **Zoom-to-rect**: This tool may be toggled on and off. When it is on, you can use the mouse to click and drag a rectangle to zoom in on. The extents of the rectangle will become the new extents of the axes. Use the Back button (below) to zoom back out.
- **Pan**: This tool may also be toggled. When on, you can use the mouse to pan the axes by clicking and dragging.
- **Forward** and **Back**: These 2 buttons will redo and undo the zooming and panning actions that you make
- **Home**: This will reset the view to the way it was after Update Chart was pressed.

> ***Note***
>
> Update may take a long time for large databases. We are working to improve this performance in later releases.

XII. Box Plot

Launch **Box Plot** from the main CPA interface by clicking the button in the toolbar. This tool will allow you create box and whisker plots from the numeric columns in your tables.

To use the **Box Plot**, simply select the table whose column(s) you would like to plot from the table dropdown. Then select the measurement you would like to plot from that table in the x-axis dropdown (only numeric columns will be available). To plot multiple measurements against the same y-axis, click the **Select Multiple** button to the right of the x-axis dropdown. You will be presented with a checklist of columns to include in the plot. The filter dropdown can be used to select filters defined in your properties file to limit the points being plotted. Finally, click the **Update Chart** button to view the plot.

The bottom and top of the box represent the 25th and 75th percentile of the data, with the median value represented by a horizontal red line. The whiskers on the top and bottom extend to the most extreme data point within 1.5 times the upper and lower quartiles. Outliers are represented individually with “+” shapes.

Once data is plotted, you can use the tools provided in the toolbar at the top of the window to explore the plot. Going backwards from right to left, the tools are as follows:

- **Save**: Clicking this will give you choices for saving the plot as an image.
- **Zoom-to-rect**: This tool may be toggled on and off. When it is on, you can use the mouse to click and drag a rectangle to zoom in on. The extents of the rectangle will become the new extents of the axes. Use the Back button (below) to zoom back out.
- **Pan**: This tool may also be toggled. When on, you can use the mouse to pan the axes by clicking and dragging.
- **Forward** and **Back**: These 2 buttons will redo and undo the zooming and panning actions that you make
- **Home**: This will reset the view to the way it was after Update Chart was pressed.

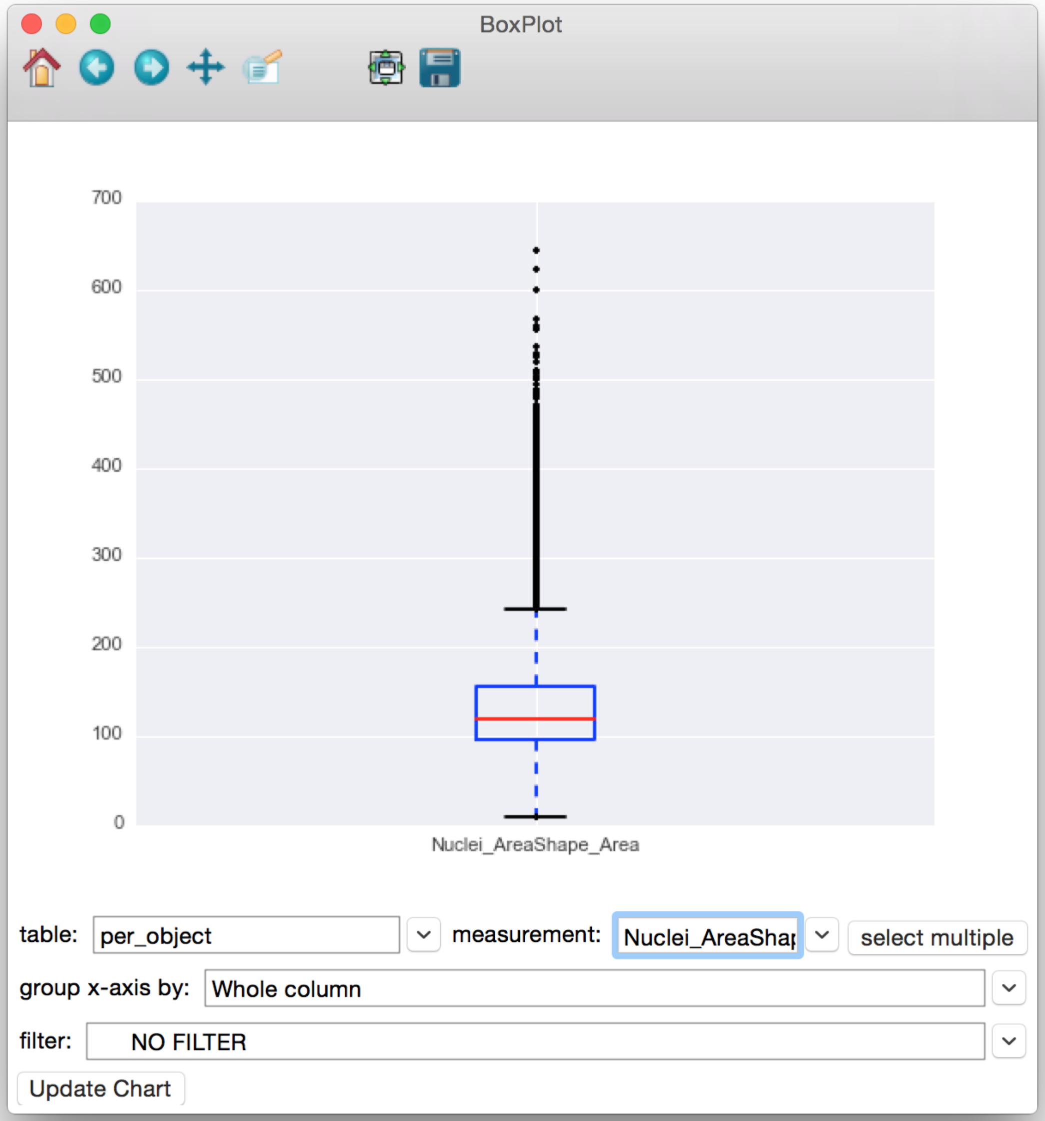

A box plot of Nuclei_AreaShape_Area.

XIII. Workspaces

Workspaces in CPA are a way of saving the state of your plots so they can be reopened later and even applied as a template to new datasets.

Suppose you are performing quality control on a biological screen in which new plates (or slides) are imaged every week. The way you process and perform quality control on each new plate is largely the same. For example, you first run a CellProfiler pipeline producing various QC measures such as focus scores and stain intensities. Then, in CPA, you want to create (for example) a DNA content histogram and a scatterplot of Measurement_X vs. Measurement_Y while filtering for your controls. You also display Measurement_Z in P**late Viewer** to look for wells that may be out of focus. All of these plots can be saved in a workspace file by CPA, and 42 applied to new data later.

To create a workspace, simply open and configure the plots that you wish to save. Then choose **File > Save workspace** from the CPA file menu. The file that you save will contain configuration details for all of the currently open plots (Note: Table Viewer and Classifier do not yet support saving configurations). These same plots can be reopened in CPA by choosing **File > Load workspace** from the CPA file menu when the same properties file is used. To apply the workspace to a new dataset, simply open CPA with a different properties file that points to your other data, then choose **File > Load workspace** from the CPA file menu. CPA will try to apply the same settings to all of the plots that were open while using your new data.

> **Warning**: If you save a histogram plot of per_image.Measurement_X in a workspace and try to open the workspace with a dataset that doesn't have a Measurement_X column in it's per_image table, CPA will simply use the first measurement in your per_image table instead.

XIV. Image Gallery

Image Gallery provides a convenient gridview allowing an overview of images from the entire experiment. A variety of options are provided to filter images based on experiment-specific metadata, e.g., gene name, compound treatments, etc. Multiple filters can be combined to refine the search. Images can be displayed as a custom-sized thumbnail or in full resolution, and the color assigned to each channel in the image can be customized to highlight structures of interest. Individual segmented cells can be viewed for each image, and can be dragged and dropped into the Classifier window.

Image Gallery consists of a menu bar, an area for the image gallery area, and an area for the objects of segmented image. In the menu bar, you can choose what image you want to fetch. You can choose a range of images based on image ID from the experiment or a filter, all images from the experiment or a filter, or an individual image based on image ID. Once the selection is made, click **Fetch** to load the relevant images.

For each image loaded in the image gallery area, right-click to show the options. - Select **View full images of selected** or double-click an individual tile to show the object in the context of the image from which it was drawn. This launches the **Image Viewer** tool (section V). - **Select all/Deselect all** (`ctrl+A`/`ctrl+D`) selects/deselects all tiles in the bin so they can be dragged and dropped together. - **Invert selection** (`ctrl+I`) to invert your selection (that is, select all non-selected tiles in the current bin and deselect all selected tiles). - **Remove selected** (`Delete`) removes the selected tiles from the current bin. - **Fetch all objects from image** populates the objects of segmented image area below with all objects belonging to the selected image(s).

Images loaded in the area for the objects of segmented image have the same right-click options except for **Fetch all objects from image**. Tiles in this area can be dragged and dropped into the Classifier.

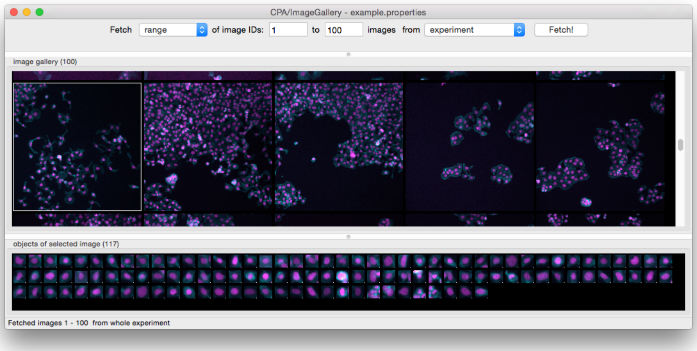

Image Gallery
