# Supplementary material for: CellProfiler Analyst: interactive data exploration, analysis and classification of large biological image sets
Source: Bioinformatics. 2016 Jun 26;32(20):3210–2. doi: 10.1093/bioinformatics/btw390 (PMC5048071; doi:10.1093/bioinformatics/btw390)
Supplement: Supplementary Data [file btw390_supplementary_data.zip › Supplementary_Data_1.doc]

# Benchmarking performance of classifiers in CPA 2.0 versus CPA 1.0

a)

b)

Fig. S1. Accuracy (a) and time (b) of a 20 fold cross validation in CPA 1.0 (FastGentleBoosting algorithm with 200 regression stumps) and CPA 2.0 (RandomForest, LDA, SVC, and LogisticRegression algorithms). Note the log scale in (b).

Goal: To compare the performance (accuracy and computational cost) of CPA 2.0 to 1.0.

Methods: The dataset used was made up of the 2526 positive example images in the 14 phenotype HT29 dataset from the Broad Bioimage Benchmark Collection (BBBC018v1) [(Ljosa, Sokolnicki, and Carpenter 2012)](https://paperpile.com/c/LJMyiJ/UbW0). Cells were stained with Hoechst 33342 (DNA), phospho-histone H3 (indicates mitosis), and phalloidin (labels actin, present in the cytoplasm).

By processing the images through CellProfiler, a set of 615 features were extracted (various measures of size, shape, texture, intensity, etc.)

Classification in CPA 1.0 is done with the FastGentleBoosting algorithm, but CPA 2.0 contains several algorithm choices from scikit-learn [(Pedregosa et al. 2011)](https://paperpile.com/c/LJMyiJ/ZPIYY), a popular machine-learning library. Among the most frequently used classifiers are RandomForest, LDA, linear SVC (a.k.a. SVM for classification), and LogisticRegression.

Accuracy and speed are benchmarked by performing a 20-fold cross-validation, where the annotated set is split into 20 parts, each part is used as a test set for the training set consisting of the other 19 parts. The accuracy and speed of each test set are averaged together to obtain the final result.

Results: Fig. S1 shows the resulting accuracy and time elapsed of a 20-fold cross-validation. Although all algorithms show a similar accuracy of ~86%, CPA 1.0’s FastGentleBoosting takes 5.5 hours to finish (data obtained from [(Abbas, Dijkstra, and Heskes 2014)](https://paperpile.com/c/LJMyiJ/VZGx)), which is 200 times slower than the slowest algorithm in CPA 2.0. This indicates the significant speed improvement of CPA 2.0, without loss of accuracy.

**References**

[Abbas, Syed Saiden, Tjeerd M. H. Dijkstra, and Tom Heskes. 2014. “A Comparative Study of Cell Classifiers for Image-Based High-Throughput Screening.” *BMC Bioinformatics* 15 (October): 342.](http://paperpile.com/b/LJMyiJ/VZGx)

[Ljosa, Vebjorn, Katherine L. Sokolnicki, and Anne E. Carpenter. 2012. “Annotated High-Throughput Microscopy Image Sets for Validation.” *Nature Methods* 9 (7): 637.](http://paperpile.com/b/LJMyiJ/UbW0)

[Pedregosa, Fabian, Gaël Varoquaux, Alexandre Gramfort, Vincent Michel, Bertrand Thirion, Olivier Grisel, Mathieu Blondel, et al. 2011. “Scikit-Learn: Machine Learning in Python.” *Journal of Machine Learning Research: JMLR* 12 (November). JMLR.org: 2825–30.](http://paperpile.com/b/LJMyiJ/ZPIYY)
